# Supplementary material for: Non‐genetic diversity modulates population performance
Source: Mol Syst Biol. 2016 Dec 19;12(12):895. doi: 10.15252/msb.20167044 (PMC5199129; doi:10.15252/msb.20167044)
Supplement: Supplementary file 1 — Appendix [file MSB-12-895-s001.pdf]

Non-genetic diversity modulates population performance

Adam James Waite, Nicholas W. Frankel, Yann S. Dufour, Jessica F. Johnston,
Junjiajia Long, Thierry Emonet

APPENDIX

Appendix Figures S1 – S4
Appendix Figure Legends S1 – S4

Non-genetic diversity modulates population performance

Figure Appendix

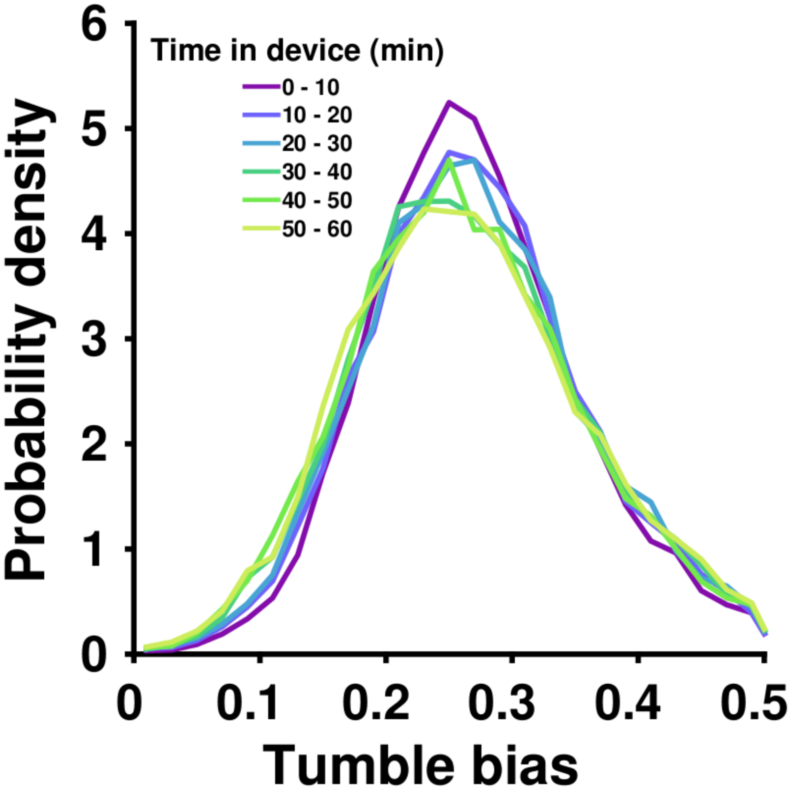

Appendix Fig. S1. Tumble bias distribution is stable in microfluidics device. A microfluidics device without a gradient of MeAsp was loaded with cells (~32,000) without engaging the gate, so that cells filled the entire chamber. The first 4.5 mm of the chamber were observed over 60 min. The tumble bias distribution of all cells in 10 min windows is shown. The mean tumble bias decreased at a rate of 1.3 $\times10^{-4}$ (95% CI: [1.27 – 1.32] $\times10^{-4}$) min^{-1}, and declined by 2.1% over the 60 minute observation.

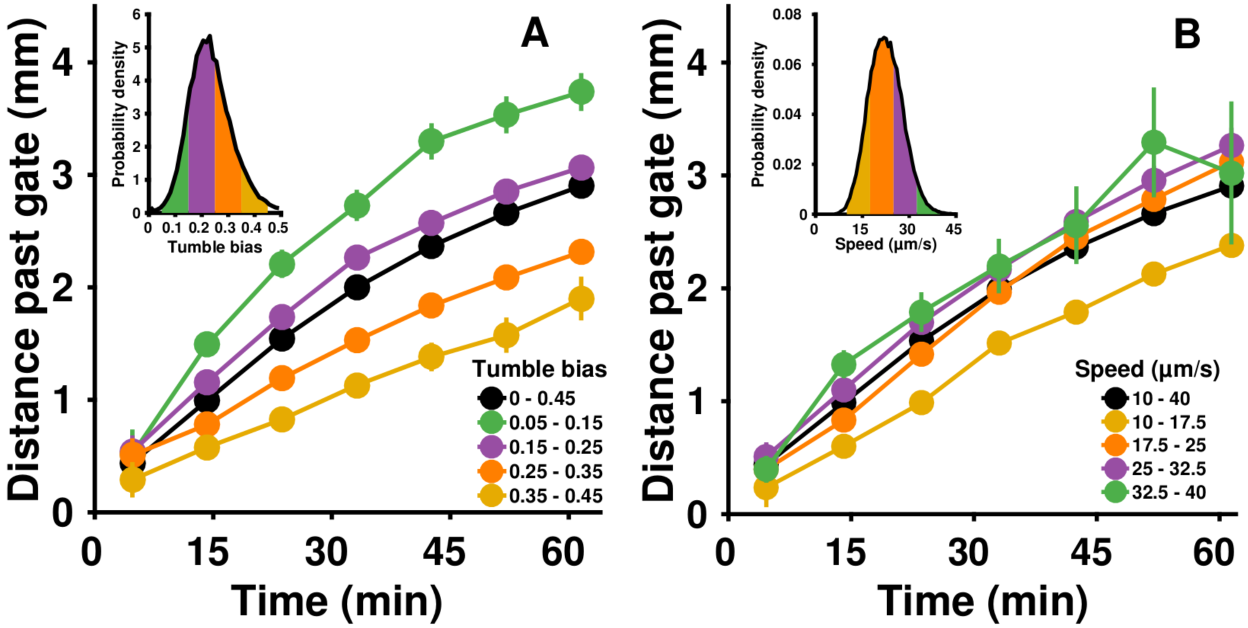

Appendix Fig. S2. Differences in position cannot be explained by differences in speed. A) Data of cells in gradient, binned by tumble bias (reproduced from Error: Reference source not foundE for comparison). **B**) Same data, binned by run speed. Error bars indicate ± two times the standard error of the mean.

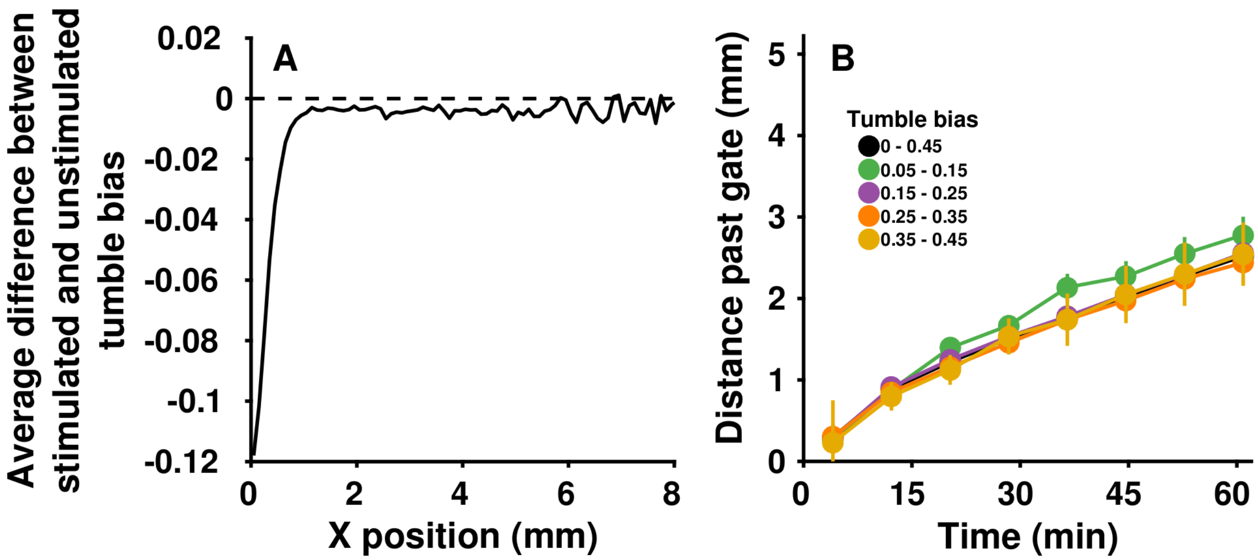

Appendix Fig. S3. The difference between unstimulated and stimulated tumble bias does not account for the observed differences in performance. A) Simulation showing the average deviation between unstimulated and stimulated tumble bias for initially identical cells. The unstimulated value was determined by simulating initially identical cells evenly distributed in the device without a gradient. **B**) The performance of the initially identical cells shown in **A** binned by their observed tumble bias.

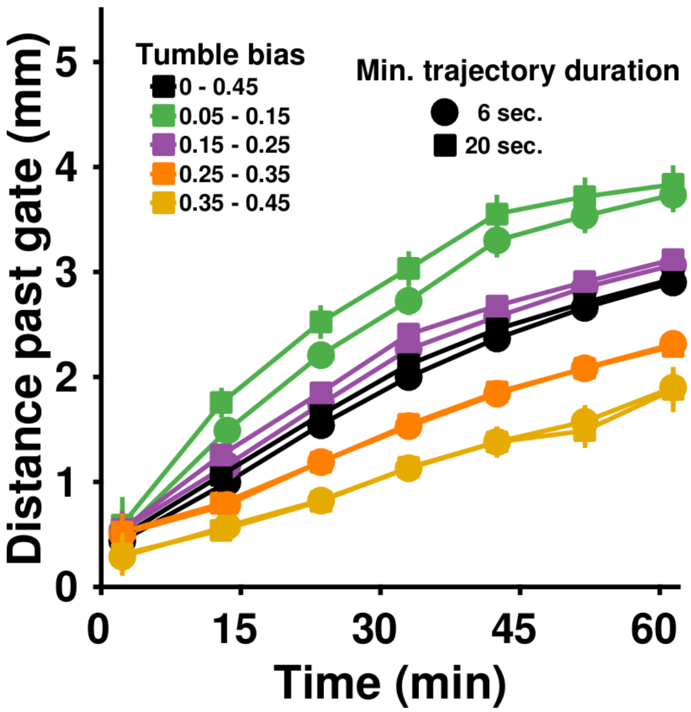

Appendix Fig. S4. The effect of increasing minimum track duration. Tracks for the wild-type population in a MeAsp gradient were analyzed with a minimum trajectory length of 6 seconds (circles) or 20 seconds (squares).
