# Supplementary material for: Non‐genetic diversity modulates population performance
Source: Mol Syst Biol. 2016 Dec 19;12(12):895. doi: 10.15252/msb.20167044 (PMC5199129; doi:10.15252/msb.20167044)
Supplement: Supplementary file 2 — Expanded View Figures PDF [file MSB-12-895-s002.pdf]

Expanded View Figures

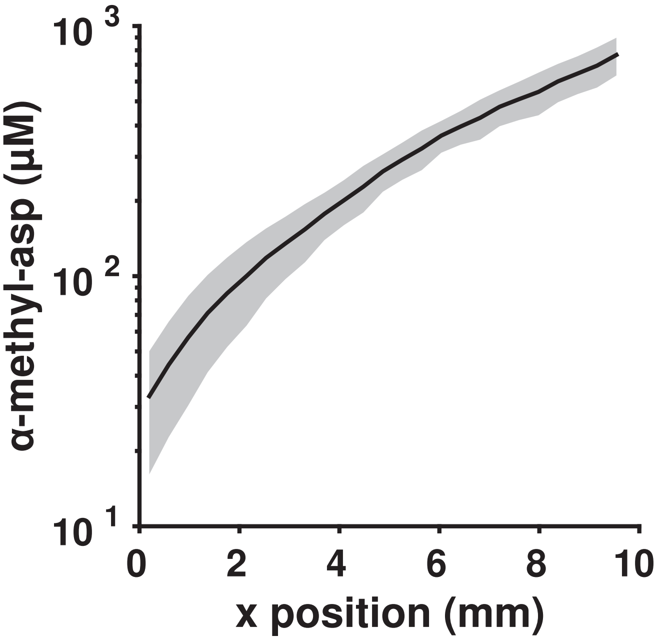

Figure EV1. The gradient profile.
The gradient profile averaged over time and across the four wild-type experiments. The gray shaded region is ± 2 standard deviations of the time-averaged mean across the four experiments.

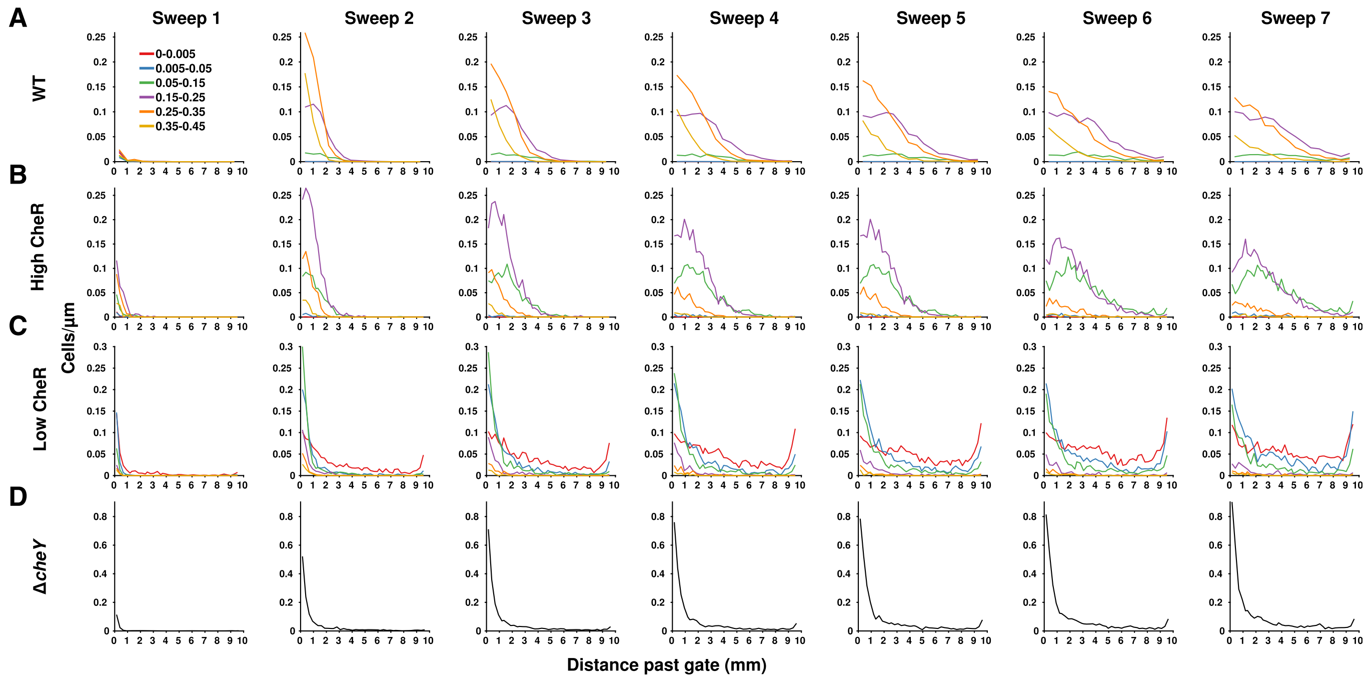

Figure EV2. Full spatial distributions of induced and Δ*cheY* strains.

A–D Cell density as a function of distance past the gate for the wild-type strain (A), and mutant strain induced with 100 µM IPTG (B), 10 µM IPTG (C), and the Δ*cheY* strain (D). Each "sweep" indicates data compiled from one sink-to-source set of movies. The approximate centered times for each sweep were 4.6, 14.0, 23.6, 33.1, 42.5, 52.0, and 61.4 min, respectively.

© 2016 The Authors

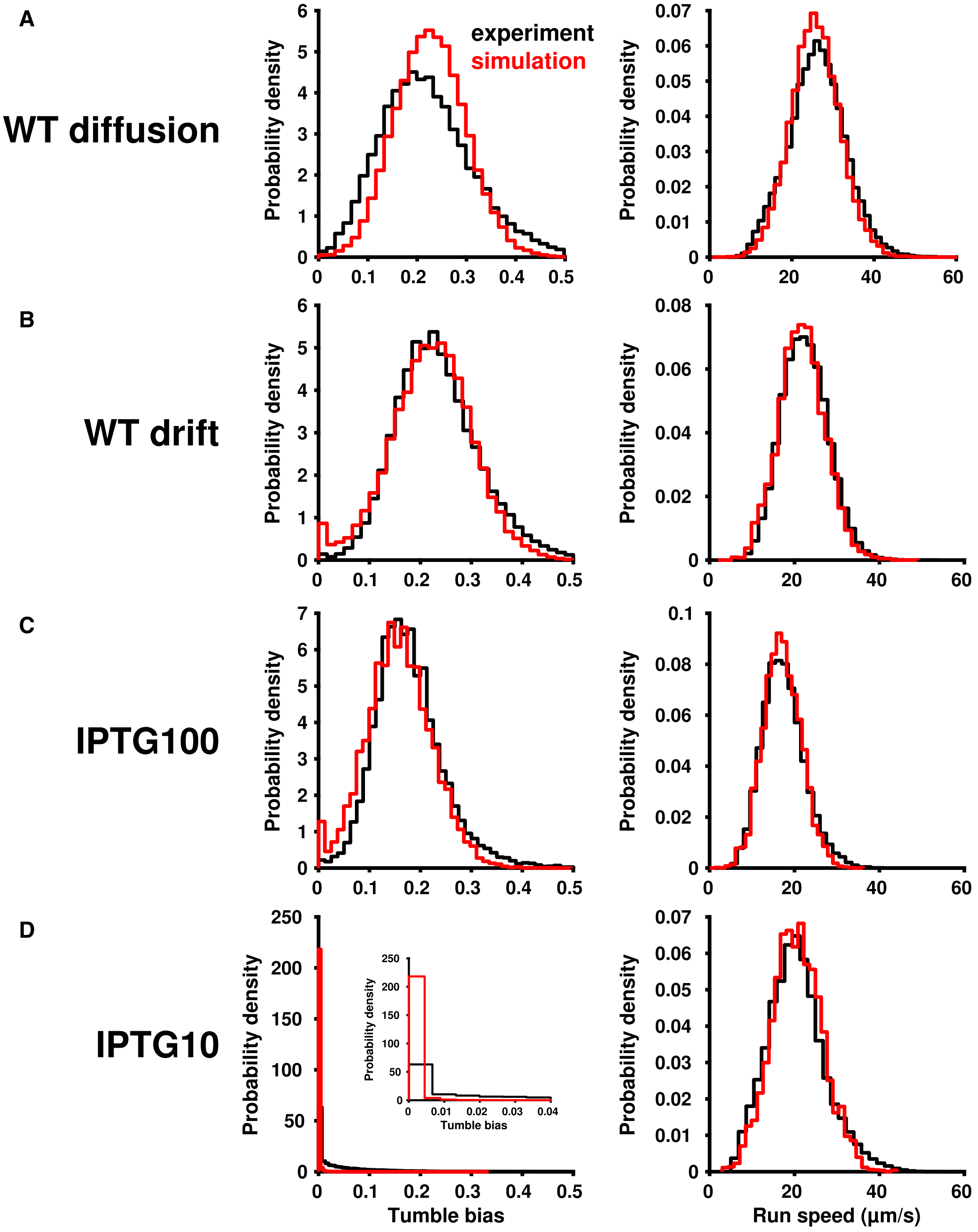

Figure EV3. Comparison of experimental and simulation phenotypes.

A–D Distributions of tumble bias (left) and run speed (right) in experiments (black) and simulations (red) for wild-type cells without (A) or with (B) a gradient, and mutant strains in a gradient induced with 100 μM (C) or 10 μM (D) IPTG. Inset of (D) shows the same data as (D) for a subset of low tumble bias. Tumble bias distributions of the simulated cells (left, red) were matched to the experimental tumble bias distribution (left, black) by altering the mean number of CheR proteins expressed per cell. Following previous reports (Li & Hazelbauer, 2004), a mean of 140 molecules/cell was used for wild-type diffusion (A) and drift (B). For the mutants, we used a mean of 120 molecules/cell for induction with 100 μM IPTG (C) and a mean of 13 molecules/cell for induction with 10 μM IPTG. Speed distributions of the simulations (right, red) were normally distributed with the following means ± SDs: (A) 30 ± 7 μm/s; (B) 26 ± 6 μm/s; (C) 20 ± 5 μm/s; (D) 21 ± 6 μm/s.

 © 2016 The Authors

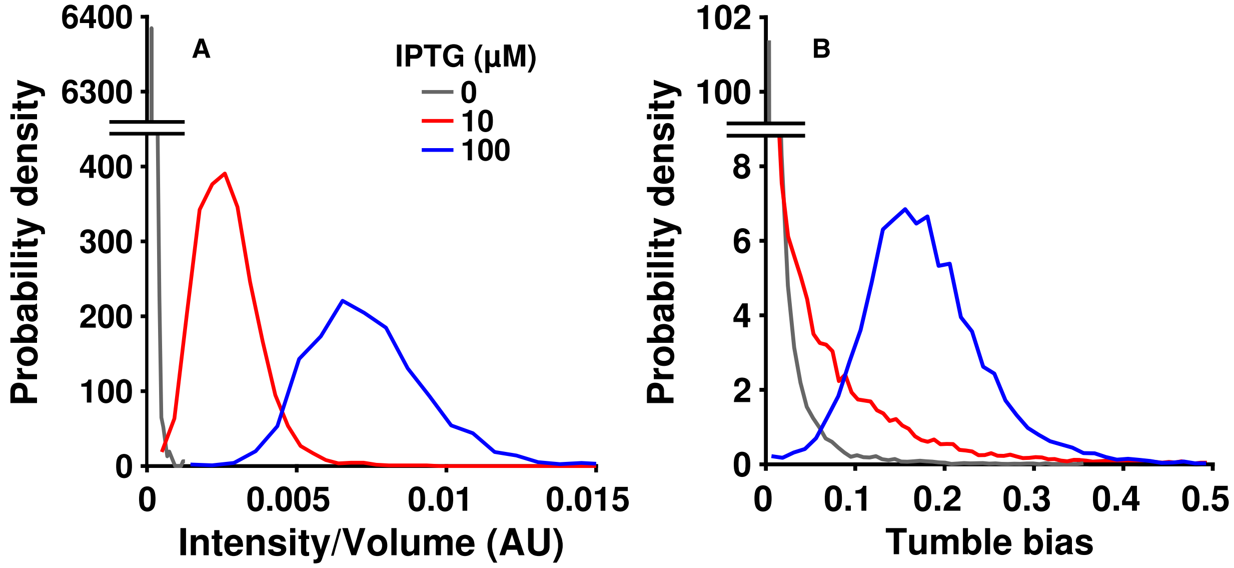

Figure EV4. Expression of CheR correlates with tumble bias.

A Populations of cells were induced with 0 (gray), 10 (red), or 100 (blue) μM IPTG, washed, and imaged on an agar pad at 100× magnification.

B Tumble bias distributions of cell populations induced with 0 (gray), 10 (red), or 100 (blue) μM IPTG as observed in the microfluidic device. Data used to make the 10 and 100 μM IPTG tumble bias distributions are reproduced from Fig 3A and B for comparison.

Data information: The distributions were constructed using 2,400, 2,799, and 2,734 cells for 0, 10, and 100 μM IPTG induction, respectively.

© 2016 The Authors

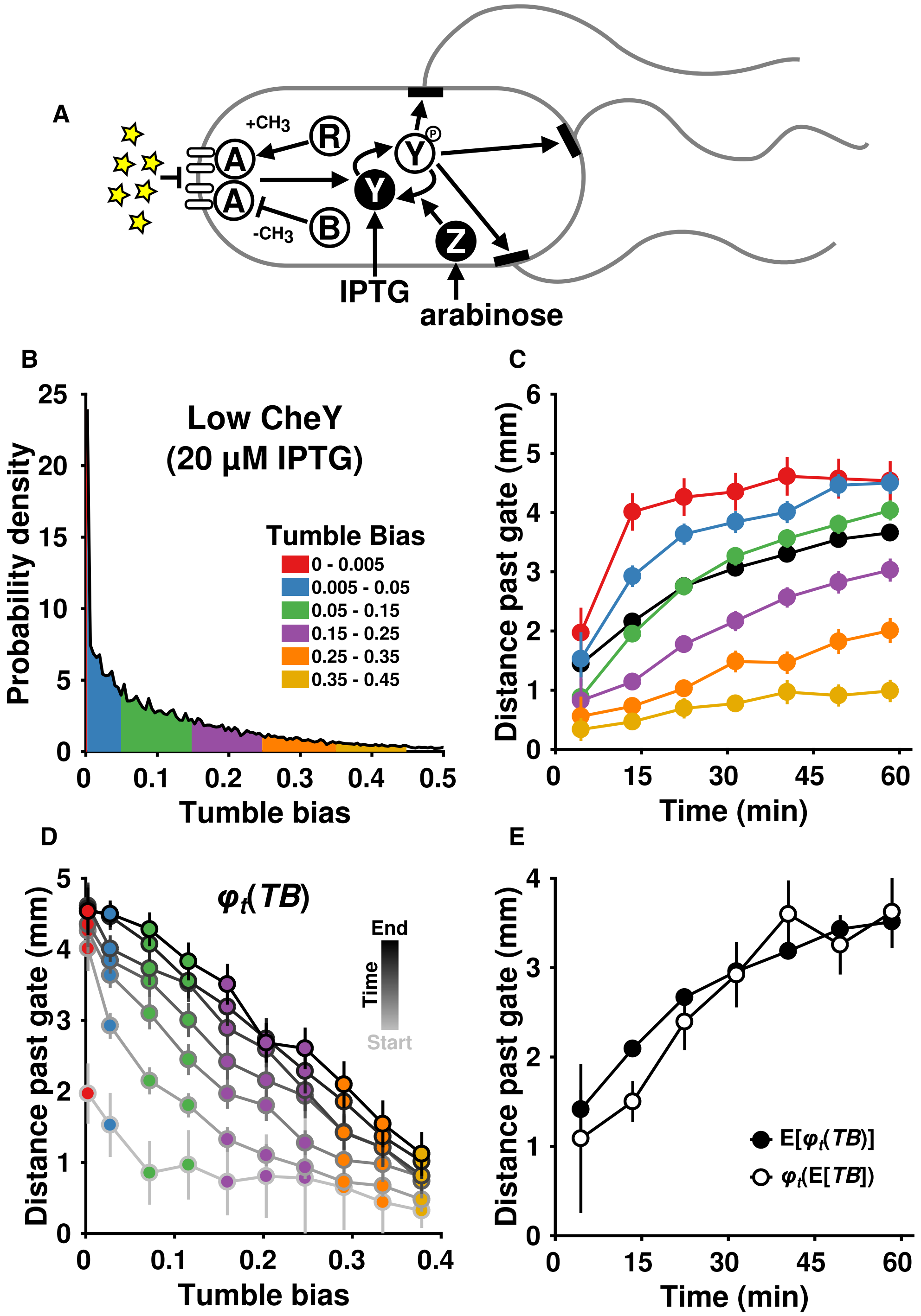

Figure EV5. Performance depends on tumble bias even when tumble bias is modified independently from adaptation time.

A Schematic of the dual-inducible CheY/CheZ strain. Symbols are same as in Fig 3A.

B Tumble bias distribution of the dual-inducible strain induced with 20 µM IPTG and 0.0001% arabinose.

C The performance of the dual-inducible strain. Black indicates the average position of the entire population.

D Performance (distance past the gate) as a function of phenotype (tumble bias, *TB*) for every pass through the microfluidics chamber was used to create the function $\varphi_t(TB)$. The lowest tumble bias point is 0.0025.

E The mean performance of the population (closed circles) and the performance of the mean phenotype (open circles) over time. The performance of the mean phenotype was defined as the average performance of cells having a tumble bias within 0.01 of the population mean tumble bias (0.13 ± 0.002). The data are from two experiments totaling 17,400 cells.

Data information: All errors and error bars indicate ± two times the standard error of the mean.

 © 2016 The Authors
